# Projected 21st century compression of mesopelagic habitat in the California current

**DOI:** 10.1038/s41598-025-10992-1

**Published:** 2025-07-22

**Authors:** Ilysa S. Iglesias, Jerome Fiechter

**Affiliations:** 1 Ocean Sciences Department, University of California, Santa Cruz, 1156 High Street, Santa Cruz, CA 95064 USA; 2 Scripps Institution of Oceanography, UC San Diego, 9500 Gilman Drive, 92093 La Jolla, CA USA

**Keywords:** Twilight zone, Mesopelagic-epipelagic coupling, Climate change, Downscaled climate projections, Regional ocean modeling, Deoxygenation, Ocean sciences, Physical oceanography, Physical oceanography, Climate-change ecology, Climate and Earth system modelling, Projection and prediction

## Abstract

Although the mesopelagic zone occupies a substantial volume of the world’s oceans, our results suggest that the livable portion may compress vertically by ~ 40 m or ~ 39% by the end of the century. Using an ensemble of three downscaled climate projections from a high emissions scenario, we evaluated the connection between anthropogenic greenhouse gas emissions and changes in light and oxygen at depth, which influence the upper and lower limits of mesopelagic habitat in the central California Current. Although the model projects a small deepening (~ 2 m) of the upper light boundary consistent with increased stratification and reduced upper ocean productivity, the main driver of vertical mesopelagic habitat compression is the significant shoaling (by ~ 44 m) of the hypoxic boundary over the course of the 21st century. Differences in dissolved oxygen across ensemble members highlight the potential influence of equatorial dynamics and the California Undercurrent in constraining the future availability of mesopelagic habitat along the U.S. west coast. Mesopelagic ecosystems connect the surface ocean to the deep sea, and a projected decrease in the vertical extent of mesopelagic habitat could have cascading effects on a broader range of marine ecosystem processes and carbon export.

**Supplementary Information:**

The online version contains supplementary material available at 10.1038/s41598-025-10992-1.

## Introduction

The deep pelagic ocean comprises up to 90% of the habitable volume of earth^1^. The upper portion of the deep pelagic ocean, the mesopelagic zone, occurs at depths between ~ 200–1000 m and is home to a diverse assemblage of life, whose vertical movements connect the surface ocean to the deep sea. In this central position between the surface and deep ocean, mesopelagic habitats (and the animals that inhabit them) are also important for biogeochemical cycling and mediate the transport of organic materials^2^. Unlike other marine habitats delineated by physical structures (corals, seafloor, coastline, etc.), mesopelagic habitats are defined exclusively by dynamical oceanographic conditions and processes^3^. Although the mesopelagic zone is vast, deoxygenation is predicted to cause a global shoaling of mesopelagic habitats, with predicted losses in biodiversity^4,5^. Deep pelagic communities are typically diverse^6^ and critical for ecosystem function; providing prey for higher trophic level predators^7^ and facilitating the vertical export of organic matter^8–11^. Although the mesopelagic zone remains poorly understood^6,12^ recent advances reveal the interconnected and substantial role mesopelagic organisms such as fishes and zooplankton play in carbon export and sequestration^10^ so any changes limiting their vertical distribution in the future could have cascading effects on the greater ocean ecosystem and carbon sequestration.

The diverse assemblage of organisms inhabiting mesopelagic depths, referred to collectively as the Deep Scattering Layer (DSL), was originally identified by their strong backscatter observed in acoustic measurements^13^. Although the DSL is a global feature of the world’s oceans, faunal communities vary horizontally relative to prevailing oceanographic conditions^3^ including primary production and temperature^4^. For diel vertical migrators, who migrate vertically to feed in productive surface waters, typically at night, and return to mesopelagic depths during the daytime to avoid visual predators^14^ the minimum depth occupied during the day has consistently been linked to light^15–21^. The lower boundary of the DSL has been linked to primary productivity and wind stress^4^ and is likely limited by physiological constraints related to dissolved oxygen^22–26^. Previous work within the California Current has demonstrated that mesopelagic fishes are constrained vertically by light and oxygen^25^ and instances of declining mesopelagic fish abundance have been linked to declines in dissolved oxygen, where it was proposed that fishes were unable to descend deep enough to evade predation from visual predators^27^. Although few studies have examined long-term trends in the vertical distribution of the DSL, a deepening by ~ 100 m was previously linked to a large marine heatwave off the US West coast (2015–2016) that increased the amount of light reaching mesopelagic depths^28^. Thus, although the upper and lower extent of mesopelagic habitat has been linked to dynamical oceanographic conditions, it is unclear how climate change impacts may alter the availability of mesopelagic habitat by the end of the century.

The California Current Ecosystem (CCE), a productive eastern boundary region driven by seasonal upwelling along the western margin of the north American continent^29^, is also the northward extension of a large oxygen minimum zone (OMZ) spanning most of the eastern Pacific and contributing to some of the shallowest observed distributions of mesopelagic communities globally^21^. In the California Current, hypoxic conditions frequently occur at depths of ~ 400 m^30^, already potentially constricting the vertical extent of pelagic fauna. The depth of the hypoxic boundary can vary seasonally in response to coastal upwelling^31^ leading to increasingly widespread hypoxic conditions in coastal regions of the northern California Current, especially during spring^32,33^. Dissolved oxygen concentrations also vary on a decadal basis, as evidenced by the period from 1984 to 2006, when mid-water oxygen at 300 m declined by 21% and the hypoxic boundary shoaled by up to 90 m in southern California^34^. Globally, continued expansion of OMZs since the mid 20th century has been linked to warming ocean temperatures (which controls oxygen saturation), stratification (which limits mixing), and changes in circulation (which influence source water and ventilation rates)^30,35^. Declining oxygen concentrations and the associated expansion of OMZs are expected to have profound impacts on biological communities^36^ through habitat compression and biodiversity loss^5^ but the specific compression effects on the mesopelagic community of the central California Current are not well studied.

Despite technological advances that have allowed for more continuous sampling of deep ocean environments, observational data characterizing the mesopelagic zone are still severely lacking^37^. While satellite measurements have vastly expanded the availability of ocean data globally, they are constrained to the near-surface layer of the ocean and therefore do not provide a solution for under sampling at mesopelagic depths. In the absence of continuous, region wide, in situ observations, regional ocean models are invaluable for identifying and predicting physical and biogeochemical processes shaping the mesopelagic environment. Here, we use an ensemble of high-resolution downscaled regional climate projections from a coupled physical-biogeochemical ocean model^38,39^ to provide the first assessment of spatial and temporal changes in the vertical extent of mesopelagic habitat in the central California Current by the end of the 21st century under the Representative Concentration Pathway (RCP) 8.5 high emissions scenario.

## Methods

### Downscaled regional climate projections

**Fig. 1:**
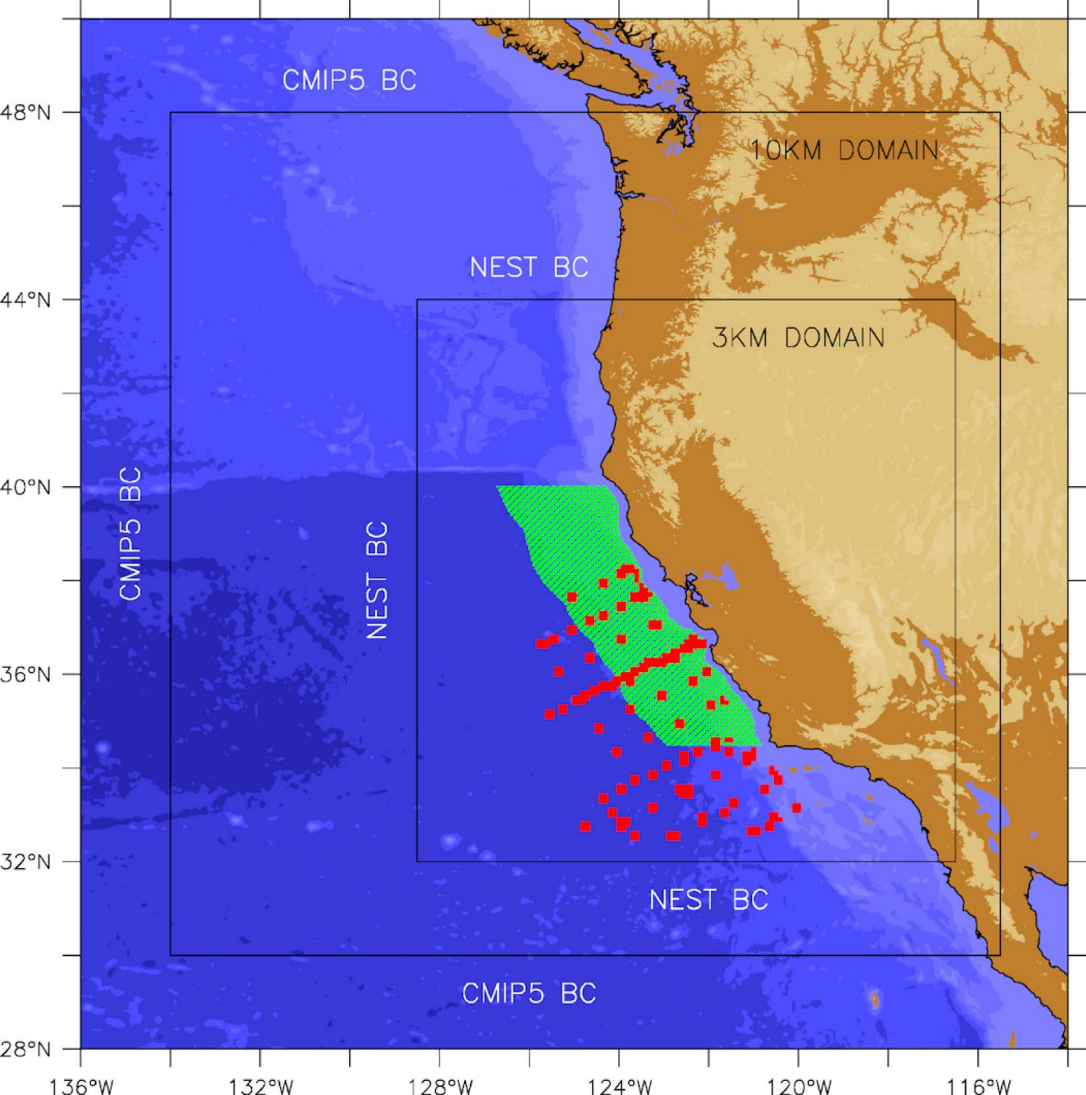
Geographical region, nested model configuration (black outlines), analysis domain (green shading), and locations of observations used for model evaluation (red symbols). The outer model domain at 10-km horizontal resolution is dynamically downscaled and forced at the open boundaries by earth system models (“CMIP5 BC”). The inner model domain at 3-km horizontal resolution is nested inside the 10-km domain and forced at the open boundaries by the downscaled projections (“NEST BC”).

We use an ensemble of high-resolution regional climate projections representing three different rates of warming under the RCP 8.5 high emissions scenario for the period 2000–2100. The earth system model solutions are first downscaled to 1/10° (~ 10 km) resolution for the broader California Current (30–48°N)^40,41^ and subsequently nested at 1/30° (~ 3 km) resolution for the central California Current (32–44°N) to improve the representation of local scale coastal upwelling processes^42^. In this approach, the 1/10° downscaled projections are forced directly by the CMIP5 earth system models using a time-varying delta method, and the high-resolution nested projections are subsequently forced by the downscaled projections using an offline nesting method (Fig. 1). The three earth system model solutions selected here are GFDL-ESM2M, IPSLCM5, and Hadley-GEM2-E as they include marine biogeochemical fields and represent the spread of the CMIP5 ensemble (GFDL-ESM2M = low rate of warming; IPSLCM5 = rate of warming close to ensemble mean; Hadley-GEM2-E = high rate of warming). While the RCP8.5 scenario may arguably depict an “unrealistically” warm future, we consider it useful here for two reasons: (1) it allowed exploring changes in mesopelagic habitat properties under extreme warming (e.g., identify potential thresholds), and (2) the lowest rate of warming (GFDL-ESM2M) is representative of the high end of the more moderate RCP4.5 scenario.

The physical and biogeochemical fields for both downscaled (1/10°) and nested (1/30°) projections are generated using an implementation of the Regional Ocean Modeling System (ROMS)^38,43^ for the California Current System coupled to NEMUCSC, a customized version of the North Pacific Ecosystem Model for Understanding Regional Oceanography (NEMURO)^39^. NEMUCSC includes three limiting macronutrients (nitrate, ammonium, and silicic acid), two phytoplankton functional groups (nanophytoplankton and diatoms), three zooplankton size-classes (microzooplankton, copepods, and euphausiids), three detritus pools (dissolved and particulate organic nitrogen and particulate silica), as well as carbon and oxygen cycling^44,45^. The three high-resolution nested projections of the coupled ROMS-NEMUCSC model are hereafter referred to as “ROMS-GFDL”, “ROMS-IPSL”, and “ROMS-HADL”.

### Evaluation of downscaled projections

**Fig. 2:**
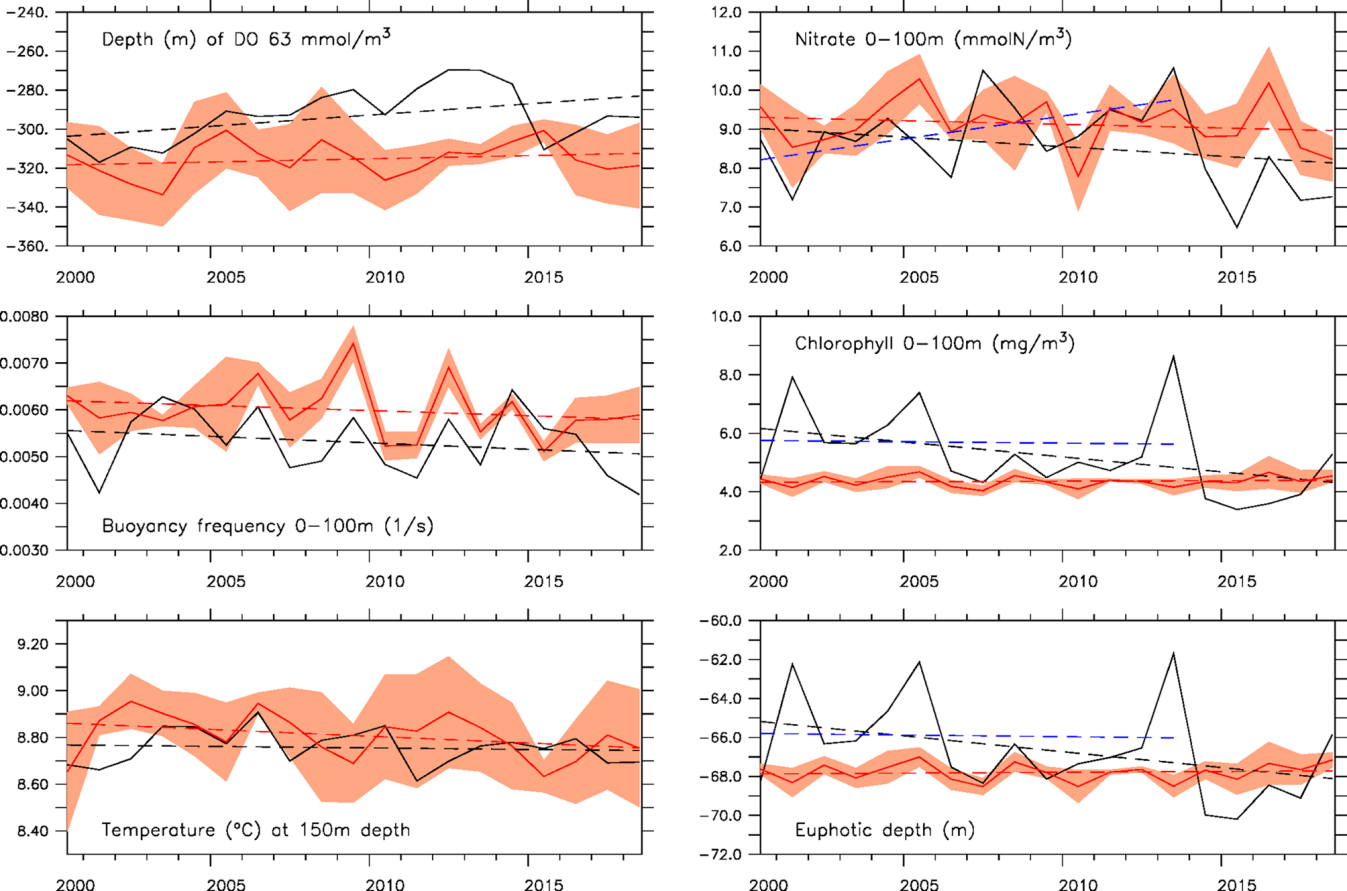
Model evaluation for present-day (2000–2020) period. Left panels: depth (m) of 63 mmol/m3 dissolved oxygen value (top), 0–100 m peak buoyancy frequency (1/s) (middle), temperature (°C) at 150 m (bottom). Right panels: 0–100 m average nitrate concentration (mmolN/m^3^) (top), 0–100 m integrated chlorophyll concentration (mg/m^3^) (middle), depth (m) of euphotic zone (bottom). In all panels, the solid black line represents observations, the solid red line represents the ensemble mean, the orange shading represents the ensemble spread, and the dashed lines represents the ensemble mean (red) and observed (black for 2000–2020; blue for 2000–2013) trends.

The high-resolution nested implementation of the coupled physical-biogeochemical model has been extensively evaluated for its ability to represent the recent historical variability of physical and biogeochemical fields (including krill, dissolved oxygen, and carbonate system) in the central California Current^42,44–46^. Hence, model evaluation was focused here on the performance of the three projections (ROMS-GFDL, ROMS-IPSL, and ROMS-HADL) during the 2000–2020 period for which concurrent observations of temperature, salinity, nitrate, dissolved oxygen, and chlorophyll over the model domain are available from the Multistressor Observations of Coastal Hypoxia and Acidification (MOCHA) synthesis product^47^. Since earth system model solutions represent a free-running evolution of the climate system, they are only expected to reproduce past variability in a statistical sense (e.g., an earth system model will have El Niño events, but not necessarily during years when actual El Niño events were observed). Due to the inherent lack of temporal correlation between the projections and observations, the model solutions were evaluated in terms of means, biases, and trends relative to observed values (Fig. 2). We also focus on ocean properties relevant to the mesopelagic zone analysis (Table 1). Prior to comparison, the projections were first sampled at the locations and times of available observations within the nested model domain (Fig. 1), and both were subsequently averaged spatially and annually to produce a series of yearly simulated and observed values (from which means, biases, and trends were then calculated). To conform with the notion that the three projections should not be analyzed as individual solutions but rather as an ensemble, the model-data comparisons were focused on relating observed properties to the ensemble mean and spread of the projections over the 2000–2020, historical period.

### Evaluating changes in the vertical extent of mesopelagic habitat

**Fig. 3:**
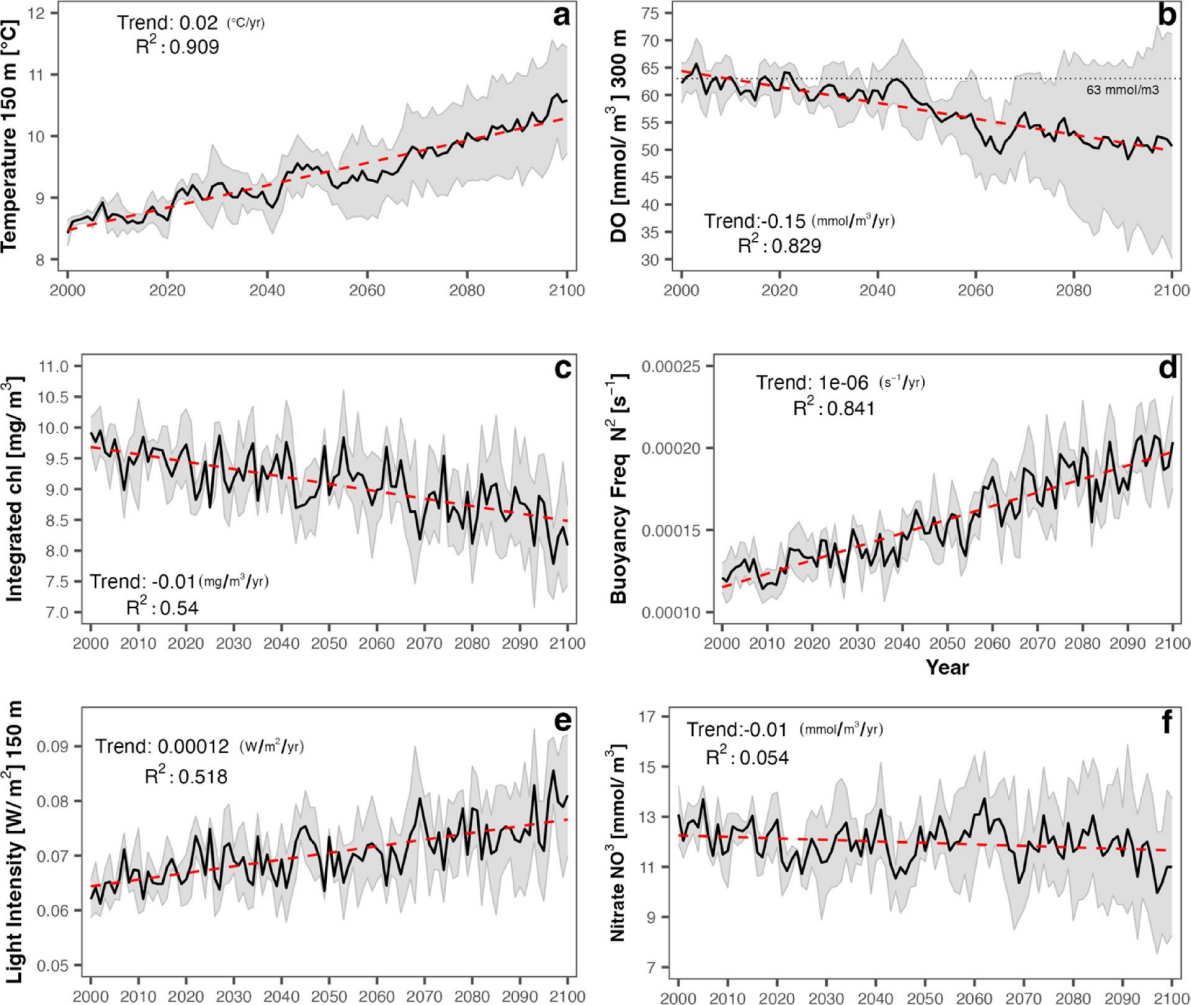
Ensemble mean (solid line), spread (shaded area) and linear trend (red) for spatial means of (**a**) temperature at 200 m, (**b**) dissolved oxygen at 200 m, (**c**) total chlorophyl integrated over the upper 100 m, (**d**) maximum buoyancy frequency *N*^2^ in the upper 150 m (main thermocline depth), (**e**) Light intensity at 200 m and (**f**) Mean nitrate concentration in the upper 100 m. The ensemble spread is defined as the standard deviation of the three ensemble members (ROMS-GFDL, ROMS-IPSL and ROMS-HADL), relative to the ensemble mean.

**Fig. 4:**
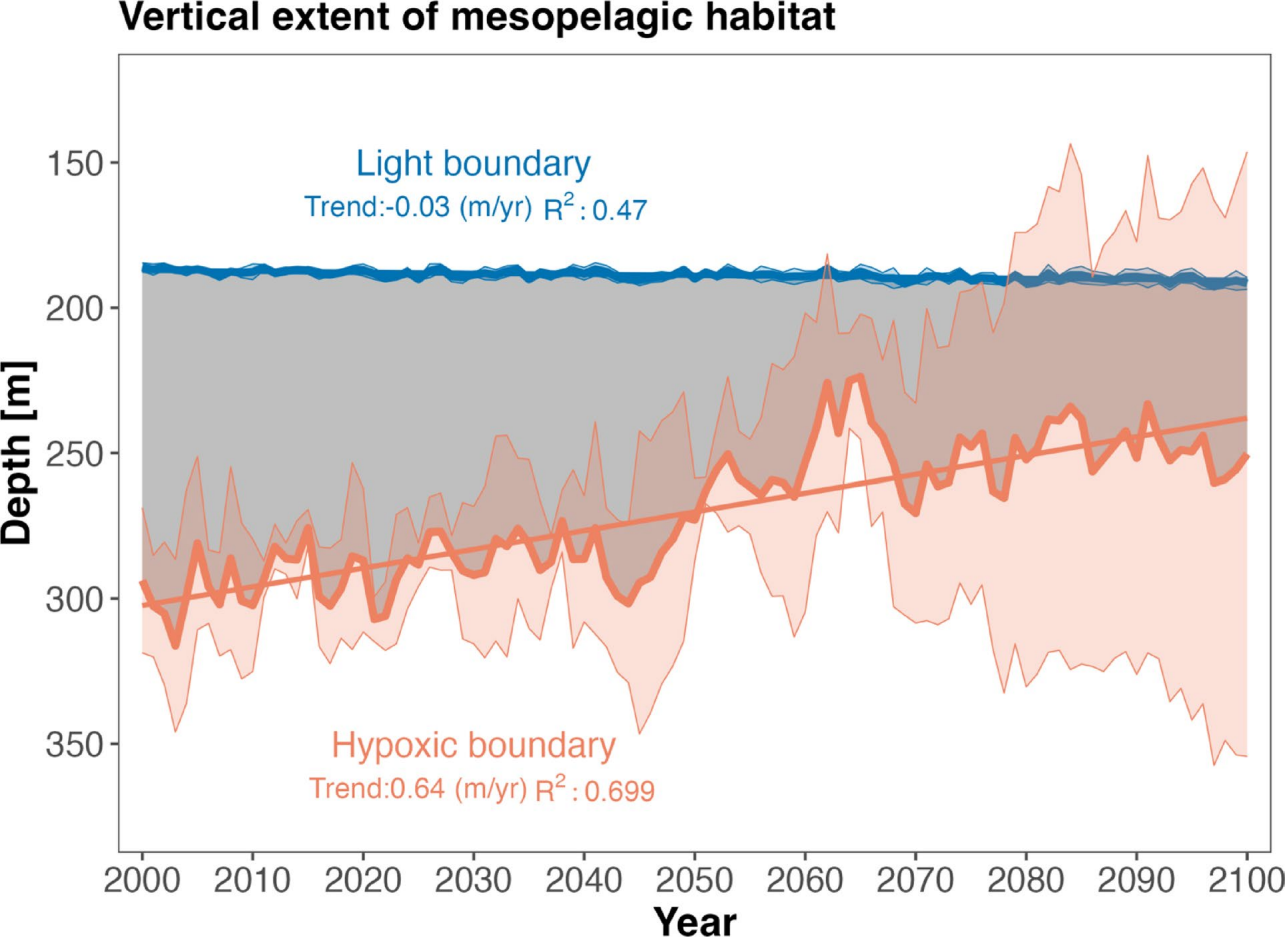
Changes in the vertical extent of mesopelagic habitat based on the spatially averaged ensemble mean and spread of three high-resolution climate projections for the central California Current. The upper boundary (blue) represents the depth at which light = 0.0217 $\:W/{m}^{2}$, a biologically relevant light threshold for animals trying to avoid visual predators. The lower boundary (orange) is the depth at which dissolved oxygen concentrations become anoxic (63 $\:\mu\:mol/l)$. The vertical extent of the mesopelagic zone (gray) is the distance between the upper and lower boundaries.

The analysis domain encompasses the latitudes from Point Conception (34.5 °N) to Cape Mendocino (40 °N) and extends longitudinally from the 400 m isobath to 200 km offshore (Fig. 1). A distance of 200 km offshore was chosen to span the core of the poleward flowing California Undercurrent^48^. A 400 m bottom depth minimum was selected to incorporate the shallower end of typical center of mass ranges for deep scattering layers estimated from acoustics within the study area^28,49^. Thus, while we include some regions located in the mid-waters above slope habitat, our analysis omits possible mesopelagic habitat shallower than 400 m bottom depths. For each oceanic metric (Table 1), we examined ensemble mean and spread of the three projections (ROMS-GFDL, ROMS-IPSL, and ROMS-HADL) (Fig. 3) over our study domain (Fig. 1). Ensemble spread, calculated as the standard deviation relative to the ensemble mean, represents a measure of model uncertainty in our ability to predict the future evolution of mesopelagic habitat characteristics due to variability in warming rates across projections under a fixed emission scenario. Light has consistently been shown to limit the upper vertical daytime distribution of mesopelagic aniamls^15–17,21,28,50^ as they retreat to darker depths to avoid visual predators. Thus, we define the upper boundary of mesopelagic habitat as the depth as which light reaches $\:{10}^{-1}\mu\:mol/{m}^{2}/s\:(\sim0.0217\:{W/m}^{2})$, the light intensity value proposed by Kaartvedt et al.^17^ as the upper limit of mesopelagic habitat, based on light sensitivities of Pearsides (*Maurolicus spp*.). The lower extent of mesopelagic habitat is defined as the depth at which dissolved oxygen concentrations reach 63 $\:mmol/{m}^{3}\approx\:63\mu\:mol/l$. This concentration was used previously to define a global hypoxic boundary^35^ and is similar to the threshold defined by Gray et al.^51^, at which mortality can occur in organisms inhabiting the water column (2.0 mg O2/L ~ 62.5 µmol/l). The vertical extent of mesopelagic habitat is calculated as the distance (in meters) between the depth where light reaches $\:0.0217\:{W/m}^{2}\:$above and the depth where oxygen reaches $\:63\:mmol/{m}^{3}$ below (Fig. 4), omitting those areas when either boundary was not reached.

**Table 1:** Oceanographic metrics used to describe changes in the mesopelagic zone by 2100.

Ocean Metric	Description
Upper boundary of mesopelagic zone: defined as the depth at which $\:Light\:intensity=0.0217\:{W/m}^{2}$	Light value proposed by Kaartvedt et al.^17^ ($\:{10}^{-1}\mu\:mol/{m}^{2}/s$ ~$\:0.0217\:{W/m}^{2}$) as the upper limit of the mesopelagic zone based on light sensitivity of Maroulicus spp. Mesopelagic animals likely descend below this depth to avoid visual predators^14^.
Lower boundary of mesopelagic zone: defined as the depth where dissolved oxygen (DO) reaches a concentration of 63 $\:mmol/{m}^{3}$ (~ 63 µmol/l from Breitburg et al. 2018)^35^ or underlying bottom depth when the oxygen threshold is not met	Hypoxia may limit the distribution of mesopelagic animals based on physiological tolerances (although it is possible that some animals are tolerant of and even select for low oxygen conditions^25,61,62^) This value is similar to concentrations that have led to mortality in mid-water animals (Gray et al.).
Temperature at 150 m	Temperature [℃] at 150 m depth. This depth was selected due to its proximity to the mean upper light boundary for 2000–2020 (~ 187 m) and thus represents warming conditions at the upper limit of the mesopelagic zone
Dissolved oxygen at 300 m	Dissolved oxygen concentration [$\:mmol/{m}^{3}]$ at 300 m. Low oxygen can limit the vertical distribution of mesopelagic animals and this depth was selected because it is close to the hypoxic boundary depth over the historical period 2000–2020 (~ 294 m).
Depth integrated chlorophyll in the upper 100 m	Simulated chlorophyll concentrations were derived from total simulated phytoplankton biomass (in $\:mmolN/{m}^{3}$) using a constant Redfield C: N of 106/16 and a Chl: C of 0.03 representative of bulk phytoplankton community properties in the southern California Current (Li et al., 2010)^73^. Chl is a proxy for phytoplankton biomass in the upper water column and has been linked to the distribution of mesopelagic communities^3,4^
Buoyancy frequency $\:{N}^{2}$	Max $\:{N}^{2}$ within the upper 100 m (main thermocline depth). $\:{N}^{2}$ is a measure of the stratification in the water column and is expected to increase with increasing ocean temperatures.
Light Intensity $\:W/{m}^{2}$at 150 m	Light can influence the vertical distribution of mesopelagic animals^15–20,25,50^. A depth of 150 m was selected because it is close to the mean upper light boundary for the historical period (2000–2020) (~ 187 m).
Euphotic zone (as a proxy for light attenuation)	The base of the euphotic zone was defined as the depth where light intensity reached 1% of the surface value and bulk attenuation was calculated using the 0–100 m depth-integrated chlorophyll concentrations and empirical k-Chl relationship derived by Li et al.^73^ for the southern California Current. The depth of the euphotic zone is independent of surface irradiance and provides a direct estimate of light attenuation in the upper water column.
0–100 m depth-average nitrate concentrations (NO_3_)	A metric to describe the availability of nitrate (an important nutrient required for photosynthesis) in the upper water column.

## Results

### Model evaluation

Model biases are relatively small (< 10% of observed mean) for most properties considered here, except for depth-integrated chlorophyll (~ 15% of observed mean) (Fig. 2, solid lines). However, the impact of the chlorophyll bias on light attenuation (i.e., related to the light metric of interest in our analysis) is substantially less important, with a resulting euphotic depth bias of ~ 2% of the observed value. Because Chl is correlated strongly with light intensity at 150 m (Pearson’s Correlation coefficient of − 0.98) (Fig. S3), we assume that our evaluation of simulated chlorophyll and corresponding light attenuation is sufficient to predict light intensity at depth, which we did not have direct measurements for. The larger bias for depth-integrated chlorophyll concentrations results in part from using a fixed Chl: C to convert from simulated phytoplankton biomass to chlorophyll, as the value of Chl: C is known to vary temporally and spatially (especially with depth). For several properties (hypoxic depth, stratification, and nutrients), the magnitude of the bias is comparable to that of the ensemble spread across the three model solutions. The direction of the observed trend is also generally respected in the simulations, but with larger discrepancies in magnitude (Fig. 2, dashed lines). For the period considered here, the ensemble mean underestimates both the shoaling of the hypoxic depth and the reduction in upper ocean nitrate concentrations by a factor of 3–4. However, robustly characterizing a long-term trend based on a 20-year period is difficult in a region strongly influenced by decadal basin-scale variability and undergoing severe, multi-year extreme events. This problem is further compounded by the fact that the projections are not guaranteed to be in-phase with each other (or with the observations), which tends to smooth out temporal variability in the ensemble mean. For example, calculating the observed change based on the years prior to the 2014–2016 and 2019 large marine heatwaves (i.e., 2000–2013) would reverse the trend for nitrate concentrations and suggest a long-term increase. This limitation also applies to chlorophyll concentrations, as evidenced by the large reduction in the magnitude of the observed negative trend when the effect of the 2014-2016 and 2019 marine heatwaves are removed. While the simulations still underpredict changes in chlorophyll concentrations and euphotic zone depth based on 2000–2013, the magnitude of the observed trend relative to the observed mean is substantially less and more comparable to those predicted by the model (< 0.2% annually for chlorophyll and < 0.02% annual for euphotic depth). Considering these caveats, but respecting model-data differences, our evaluation indicates reasonable agreement between simulated and observed values for important properties related to quantifying mesopelagic habitat but also suggests that our analysis may underpredict the magnitude of vertical compression since the projections underestimate the shoaling of the hypoxic boundary and deepening of the euphotic depth (at least based on the recent historical period).

### Projected changes in mesopelagic zone properties

The ensemble mean of the three projections (ROMS-GFDL, ROMS-IPSL, and ROMS-HADL) suggests a warmer, more stratified and less oxygenated mesopelagic zone in the central California Current by the end of the 21st century (Fig. 3). At 150 m, temperature is projected to increase significantly from 8.81 to 10.09 °C (*p* < 2.2e-16, t-test, trend: 0.02 $\:^\circ\:C/yr,\:{R}^{2\:}:0.909$), and light to increase from 0.067 to 0.075 $\:W/{m}^{2}$ (*p* = 2.173e-11 t-test, trend: 0.00012 $\:W/{m}^{2}/yr$, $\:{R}^{2}:0.52$). Dissolved oxygen concentration at 300 m is projected to decline by almost 10 $\:mmol/{m}^{3\:}$from 61.86 to 51.93 $\:mmol/{m}^{3}$ (*p* < 2.2e-16 t-test, trend: − 0.15 $\:\mu\:mol/l/yr$, $\:{R}^{2}:\:0.83$ ) (Fig. 3). The increase in light intensity near the upper boundary of the mesopelagic zone is consistent with projected decreases in mean nitrate (slight decreasing trend of − 0.0060 $\:mmol/{m}^{3},\:{R}^{2:}0.054$ and a non-significant decrease in mean from 12.06 to 11.75 $\:mmol/{m}^{3}$, *p* = 0.080 t-test) and depth integrated chlorophyll, a proxy for phytoplankton biomass (trend: − 0.01 $\:mg/{m}^{3}\:$, $\:{R}^{2}:0.54$ and a mean decline from 9.45 to 8.64 $\:mg/{m}^{3}($t-test *p* = 9.77e-13)), which could affect the amount of light attenuated in the water column. The tendency toward a less productive surface ocean (i.e., increased stratification and decreased nutrient and chlorophyll concentrations) is consistent with the projected deepening of the upper boundary of the mesopelagic zone (Fig. 4); however the magnitude of this deepening trend is small (− 0.034 m/yr, $\:{R}^{2}:$0.47), increasing only 2 m by the end of the century (from 188 m over the period 2000–2030 to 190 m from 2070 to 2100, *p* = 7.78e-11 t-test). This slow deepening of the upper boundary is in stark contrast with the rapid projected shoaling of the lower mesopelagic boundary (Fig. 4), defined by hypoxic oxygen conditions (63 $\:mmol/{m}^{3}$). This lower boundary shoaled by ~ 44 m from ~ 293 m (2000–2030) to ~ 249 (2070–2100, *p* < 2.2e-16), with a trend of 0.64 m/year ($\:{R}^{2}:0.70$).

### Projected changes in mesopelagic habitat

**Fig. 5:**
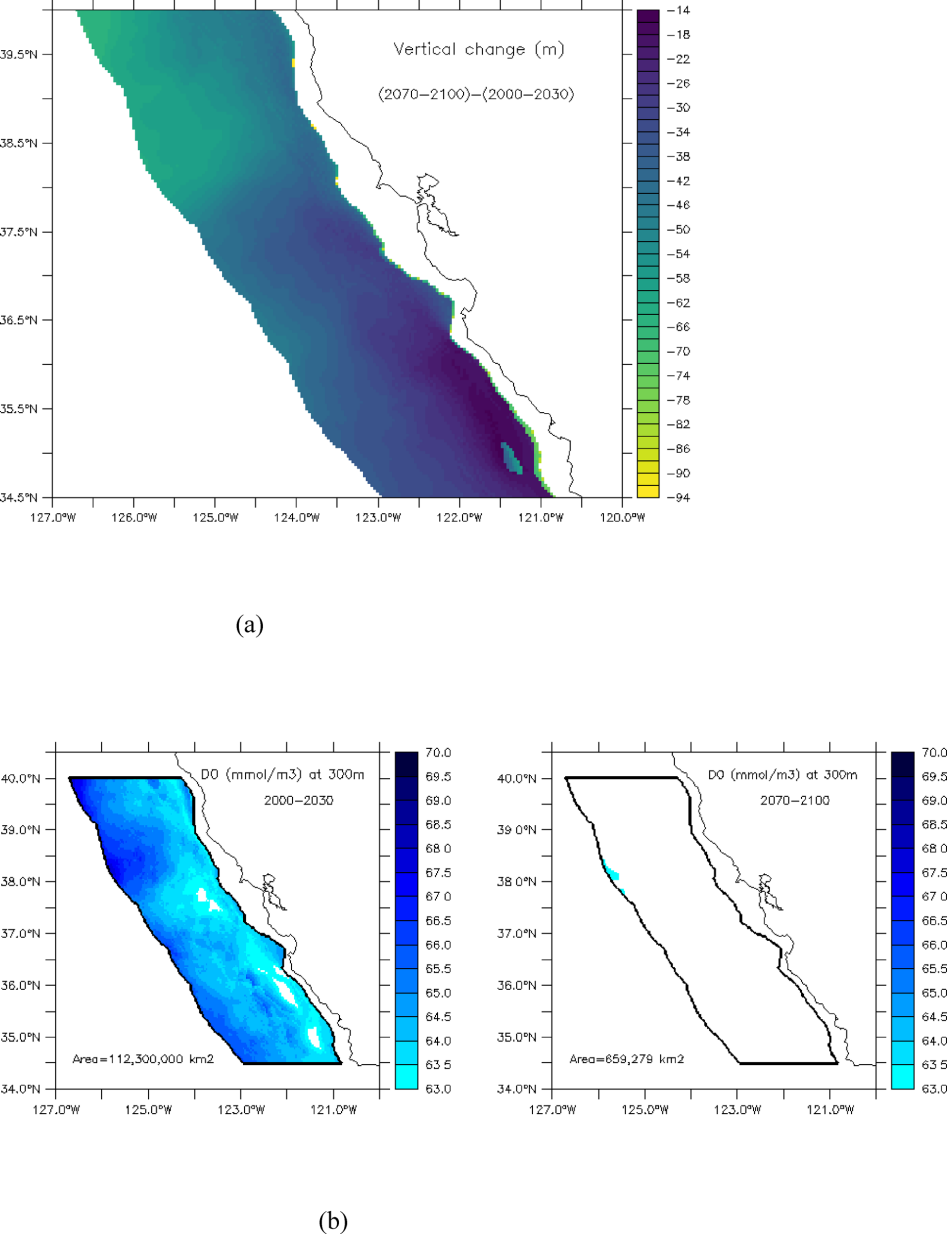
Projected changes in the vertical (**a**) and horizontal (**b**) extent of mesopelagic habitat in the central California Current by the end of the 21st century. Changes in vertical habitat represented by difference (in meters, with negative representing meters lost) between 2000–2030 and 2070–2100 (**a**). Horizontal reduction is evaluated as the extent of habitat at 300 m with DO concentrations above hypoxic threshold (> 63 $\:\mu\:mol/l$) for the beginning (2000–2030 mean, **b** left) and end (2070–2100 mean, **b** right) of 21st century

**Fig. 6:**
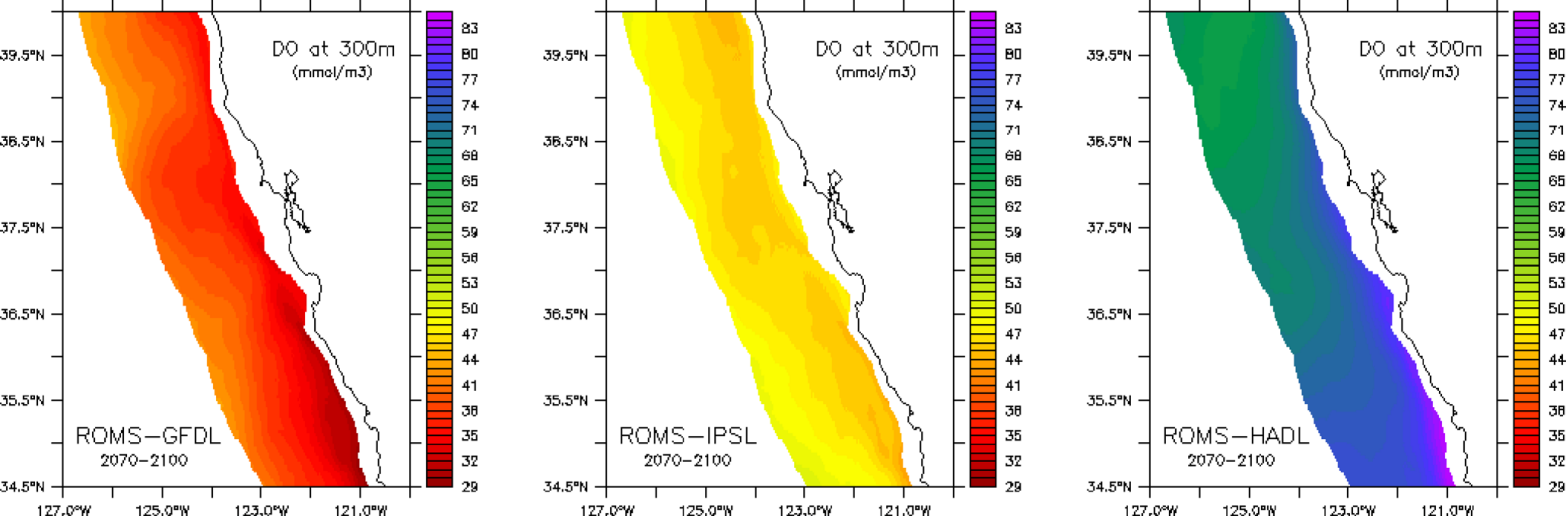
Variability in dissolved oxygen concentrations (µmol/l) at 300 m projected by the end of the century (2070–2100 means) for each ensemble member ROMS-GFDL (**a**), ROMS-IPSL (**b**) and ROMS-HADL (**c**).

The combined effects of light increasing and oxygen decling at depth yield a statistically significant decline in the vertical extent of mesopelagic habitat of ~ 41 m, or ~ 39%, by the end of the 21st century (i.e., from ~ 105 m for 2000–2030 to ~ 64 m for 2070–2100; p-value < 2.2e-16 t-test) (trend: − 0.61 m/yr, Fig. 4$\:{R}^{2}:0.70,\:$). Given that the upper boundary only deepened by ~ 2 m, most of the mesopelagic habitat decline is attributed to the shoaling of the hypoxic boundary, with the period of steepest decline occurring from ~ 2045–2065 (Fig. 4). Under the lower (ROMS-GFDL) and moderate (ROMS-IPSL) rates of warming, mesopelagic habitat declined to a mean vertical extent of just ~ 10 m (GFDL) and 22 m (IPSL) by 2070–2100 but increased to a mean of ~ 162 m under the higher rate of warming (ROMS-HADL) (Fig S2c). This divergent behavior across the three projections (Fig. 6, Fig S2) led to a substantial increase in uncertainty, as quantified by ensemble spread, in the second half of the century (Figs. 4 and 6). Furthermore, although projected ensemble means predicted loss of habitat everywhere, the magnitude of vertical mesopelagic habitat compression varied spatially, with the greatest loss occurring in the offshore region North of San Francisco to Cape Mendocino (~ 37.5 to 40 °N), and in a narrow band along the continental slope (Fig. 5a).

When translated to a loss of livable conditions at a particular depth, the projections suggest that vertical compression of mesopelagic habitat will be horizontally widespread in the central California Current region. For example, changes in dissolved oxygen at 300 m, a depth near the historical mean hypoxic boundary (~ 294 m, 2000–2020), indicate that the area of habitat above the hypoxic threshold (63 $\:mmol/{m}^{2}$) is projected to decline from a mean of ~ 112,300,000 $\:{km}^{2}$ in 2000–2030 to just 659,279 $\:{km}^{2}$, or a ~ 99% reduction in available habitat at 300 m depth (Fig. 5b).

## Discussion

The projected vertical compression of mesopelagic habitat by 39% by the end of the 21st century, which would lead to a near complete horizontal habitat loss at 300-m depth (Fig. 5b), exemplifies the direct impact of anthropogenic climate change on mesopelagic habitat availability. The ensemble of downscaled projections used here demonstrates a minimal deepening (by ~ 2 m) of the light-associated upper boundary, consistent with an increase in stratification, decrease in nitrate concentrations, and, ultimately, reduction in phytoplankton biomass. Since phytoplankton can attenuate light penetrating the upper ocean, a reduction in phytoplankton biomass could increase light intensity at mesopelagic depths and lead to a deepening of the upper mesopelagic boundary. This result is also consistent with previous observations of a deepening DSL (by up to 100 m) during a large marine heatwave in the California Current^28^. During the heatwave, reductions in chlorophyll were hypothesized to have increased light intensity at depth, which animals likely responded to by descending deeper to evade visual predators. However, if mesopelagic organisms were unable to descend deep enough to evade visual predators because of oxygen limitations at the lower boundary, it is foreseeable that mesopelagic animals may become more susceptible to predation by visual hunters, as was proposed by Koslow et al. (2011) to explain observed declines in larval mesopelagic fish abundance during periods of low oxygen in the southern California Current during 2015^27^.

Dissolved oxygen at depth is predicted to decline by 1–7% globally over the next century and beyond^30^. Oxygen minimum zones (OMZs), which typically occur at mesopelagic depths, have already expanded horizontally by millions of kilometers and are predicted to continue expanding^5,35,52^. Declining oxygen within the California Current has been associated with OMZ expansion related to surface stratification and advection^34^, changes in water mass composition^53,54^, decadal climate variability^54^, gyre-scale circulation^55^, and a combination of source water properties and remineralization^48^. While our results indicate that the 21st century rate of dissolved oxygen decline at mesopelagic depths (300 m) in the central California Current (~ 16%) could exceed the global average (1–7%), they also suggest substantial uncertainty (i.e., ensemble spread) in the depth at which hypoxic conditions are predicted to occur. Based on the 10-km downscaled projections used to force our high-resolution model domain, Pozo Buil et al. (2021) attributed this divergence in oxygen content among ensemble members to differences in Equatorial Pacific circulation^40^. Specifically, the increase in mesopelagic oxygen predicted by Hadley-GEM2-E (high rate of warming) is likely associated with a future strengthening of the Equatorial Undercurrent, which transports younger, more oxygenated waters eastward^56^ while both GFDL-ESM2M and IPSLCM5 (low and moderate rates of warming, respectively, ) predict a weakening of the Equatorial Undercurrent, which would increase the contribution of older, less oxygenated waters present in the Eastern Pacific.

Consistent dynamics were identified in the high resolution nested projections presented here whereby ROMS-HADL exhibited a coastwide increase in mesopelagic habitat (driven by increasing oxygen at depth), which intensified toward the southern boundary of our study domain and along the continental slope where water masses of equatorial origin are transported into the region by the California Undercurrent at depths of ~ 100–300 m^57–59^ (Fig. 6). In contrast, both ROMS-GFDL and ROMS-IPSL predicted coastwide declines in oxygen and a widespread reduction in mesopelagic habitat (Fig. 6, Fig. S2). The influence of the California Undercurrent and northward subsurface advection of water masses with different dissolved oxygen signatures was also confirmed by examining the southern open ocean boundaries of the downscaled and nested model domains for the three projections (Fig. S1). In particular, the southern boundary of the downscaled model domain at 30 N (which is directly forced by the three earth system models) exhibits a clear opposite sign trend for DO at 200–400 m depth between HADL (positive) and GFDL and IPSL (negative). This signal is then propagated into the high-resolution nested domain (southern boundary at 32 N) as a concentrated effect along the continental slope in the expected depth range of the California Undercurrent^60^. This result highlights a possible decoupling between the direct influence of warming and increased stratification on subsurface deoxygenation and the indirect effects associated with changes in the equatorial Pacific circulation, which could potentially mitigate oxygen loss at depth and preserve future mesopelagic habitat along the U.S. west coast. It is also worth noting that the compression of vertical mesopelagic habitat in the central California Current could end up exceeding 39% since the high-resolution projections tend to underestimate the rate of shoaling of the hypoxic boundary over the present-day period (2000–2020), although estimating long-term trends based on a 20-year period is not always reliable in a region strongly influence by decadal variability and extreme events.

The vertical compression of mesopelagic habitat described here for the California Current upwelling system is also consistent with earlier work that predicted a shoaling of the mean depth of the main DSL globally from 545 to 510 m (a difference of ~ 35 m) by the end of the century based on a biogeochemical model and the relationship between acoustic backscatter, primary production and wind stress^4^. While the authors did not include oxygen as a parameter, nor examine the lower boundary of the DSL specifically, our findings indicate that the California Current may experience a faster decline in mesopelagic habitat than predicted globally. Here we demarcated the lower boundary of the mesopelagic zone based on a hypoxic threshold (63 $\:mmol/{m}^{3}$), but it is important to note that many mesopelagic animals have evolved specific adaptations to tolerate low oxygen concentrations^61,62^ and even contribute to deoxygenation at mesopelagic depths^26^. In a previous study focused on the southern California Current region, Netburn and Koslow (2015) considered oxygen to be both a physiological constraint at the lower extent of the mesopelagic zone, and also correlated with DSL depth at the upper boundary (along with light intensity), suggesting that mesopelagic animals may actually select for low oxygen conditions^25^. The authors proposed that a rapid shoaling of oxygen at the upper boundary, and outpacing that of the lower boundary, could lead to an expansion of mesopelagic habitat in the future. However, Aksnes et al.^19^ suggest that light, which is often correlated with oxygen, was the dominant property driving the upper boundary of the DSL globally^19^. Hence, there is a clear need for future work that evaluates both the individual tolerances of mesopelagic animals to low oxygen conditions, as well as environmental drivers of DSL depth, to disentangle whether future oceans will be more or less hospitable to mesopelagic animals.

Ecologically, shoaling OMZs can change predator-prey interactions^36^. The shoaling of the lower hypoxic boundary by ~ 44 m by the end of the century suggests that deep-diving predators may be able to target mesopelagic prey more easily^63^. The existence of mesopelagic prey at the upper boundary of the mesopelagic zone makes predator foraging at these depths energetically possible^63^and small vertical shifts could make deep foraging dives less favorable. Changes in mesopelagic habitat can impact top predator populations that depend on mesopelagic animals for prey^64^ and thus compression of mesopelagic habitat could affect the foraging behavior and ultimately reproductive success of top predator populations. Horizontally, the response of mesopelagic fishes to declining oxygen has previously led to increases in the abundance of warm-water, shallower OMZ adapted species off Baja California into the southern portion of the California Current^65^. It is thus possible that warming ocean temperatures and declining oxygen at mesopelagic depths may lead to a poleward shift of mesopelagic fishes^66^ and possibly an increase in their biomass^4^. The abundance of larval Mexican Lampfish *Triphoturus mexicanus* for instance, a warm water, low oxygen affinity species, increased threefold in the southern California Current during a recent marine heatwave (2014–2016)^67^. Our results, which predict vertical compression, and associated declines in horizontal mesopelagic habitat by the end of the century, highlight the need for long-term monitoring of mesopelagic communities in the California Current to evaluate both the inherent oxygen tolerances of individual species, and the full range of climate change impacts on the vertical, latitudinal and cross-shore extent of their habitat.

Despite the importance of mesopelagic organisms to carbon export^9–11,68,69^ and as prey to higher trophic level predators^7,70^, much remains to be explored about the physical and biogeochemical processes shaping the mesopelagic zone these animals inhabit^6,12,37,71^. Few long-term studies have examined changes in mesopelagic community structure at the regional level or over long time periods, making it difficult to anticipate the true biological impacts of changing environmental conditions and habitat compression on mesopelagic communities. There is also evidence from the last 460,000 years suggesting that the abundance and diversity of mesopelagic fish communities can declined dramatically when temperatures changes by only ~ 1.5 to 2.0°C^72^ so it is not unreasonable to assume that future climate changes could have dramatic effects on contemporary mesopelagic communities. Overall, our results suggest a future in which mesopelagic habitat is vertically compressed compared to historical levels and mesopelagic communities are exposed to warmer, less oxygenated waters. This finding demonstrates the interconnection between anthropogenic greenhouse gas emissions into the atmosphere and changes in the deep sea, highlighting the pressing need to consider mesopelagic ecosystems when evaluating the impacts of climate change at global and regional scales.

## Electronic supplementary material

Below is the link to the electronic supplementary material.

Supplementary Material 1

## Data Availability

Output from the coupled physical-biogeochemical model are publicly available for download via Dryad https://doi.org/10.5061/dryad.kh18932hn. The oceanographic metrics extracted from this larger output and used in our analysis are also available as individual files at the same DOI. The R code used to analyze projections of ocean metrics and generate plots in this publication are available at Zenodo at https://doi.org/10.5281/zenodo.14042341 and consist of a .Rmd (R markdown) file that the authors ran in Rstudio74. Finally, code to produce the maps and model evaluation included in this publication was written in Ferret75 (https://ferret.pmel.noaa.gov/Ferret/) and may be available upon request from the authors.
