# Supplementary material for: Projected 21st century compression of mesopelagic habitat in the California current
Source: Sci Rep. 2025 Jul 22;15:26626. doi: 10.1038/s41598-025-10992-1 (PMC12284060; doi:10.1038/s41598-025-10992-1)
Supplement: Supplementary file 1 — Supplementary Material 1 [file 41598_2025_10992_MOESM1_ESM.docx]

**
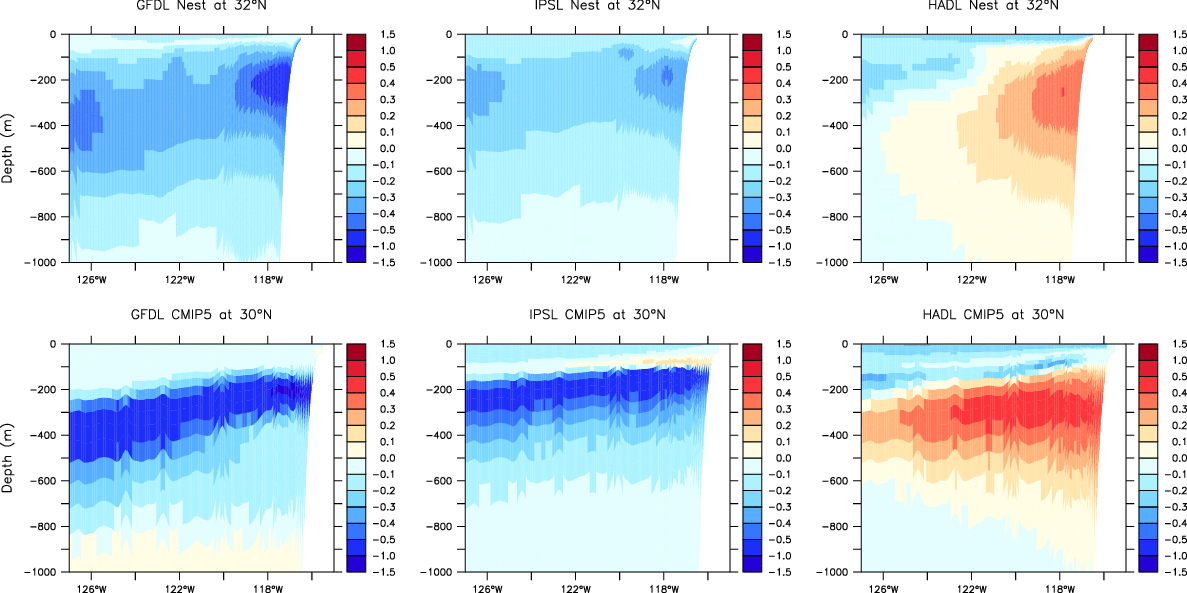
**

**Supplementary Figure 1**. 2000-2100 dissolved oxygen trend for GFDL (left), IPSL (middle) and Hadley (right). Top: southern boundary of the nested model (32N), inherited from 10km downscaled projections. Bottom: southern boundary of the 10km downscaled projections (30N), inherited from CMIP5 earth system models.


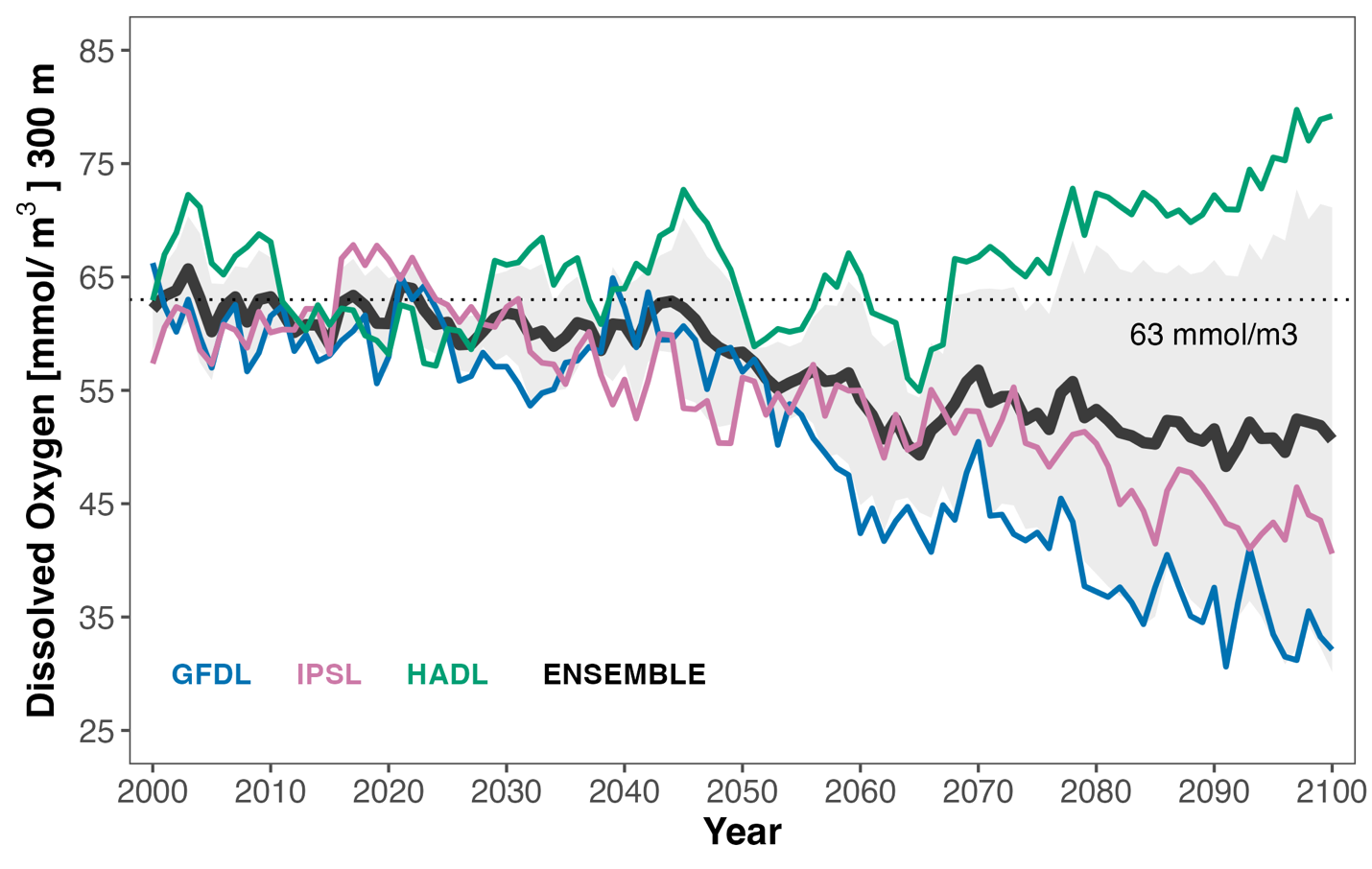


**a**


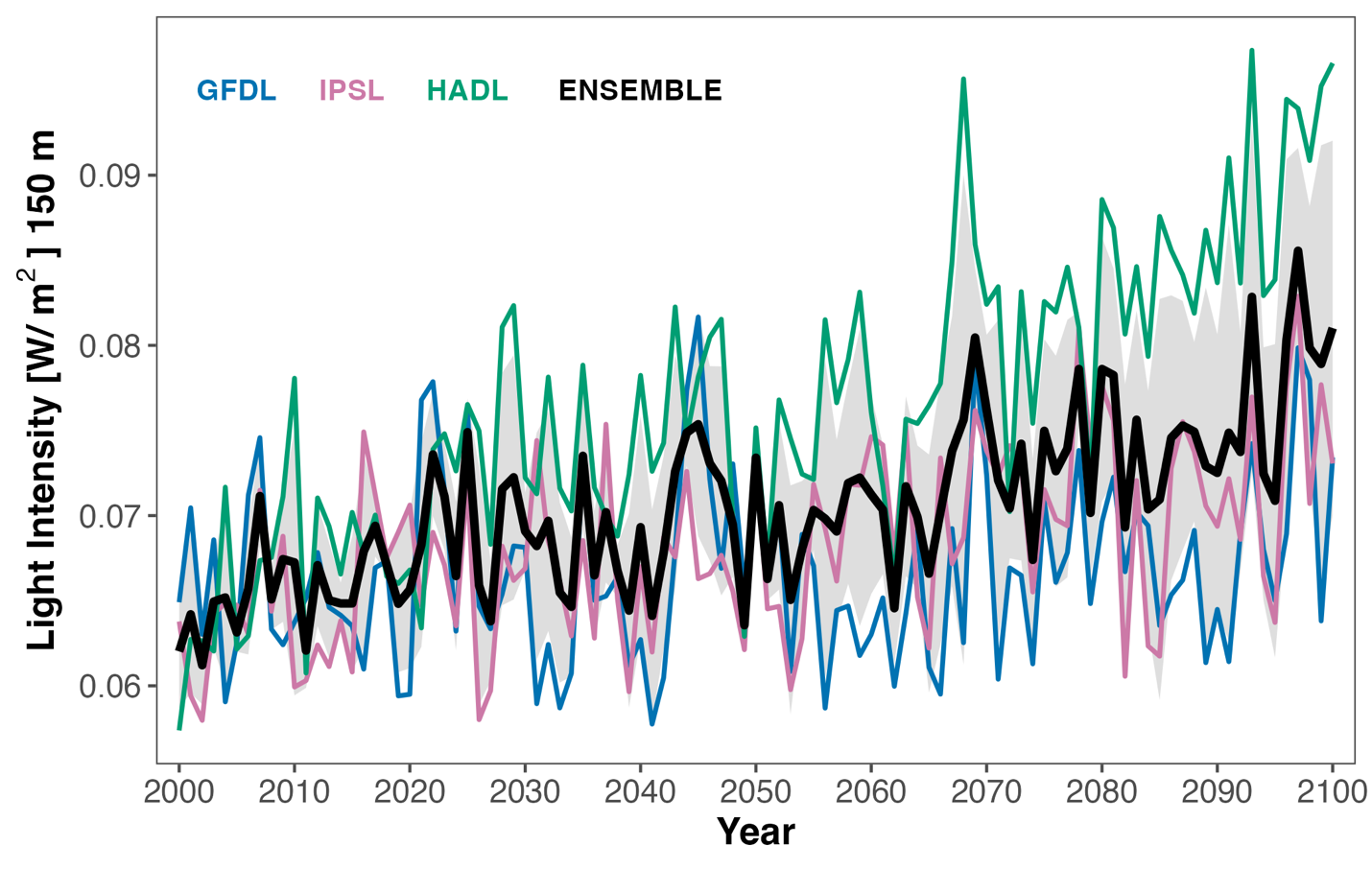


**b**


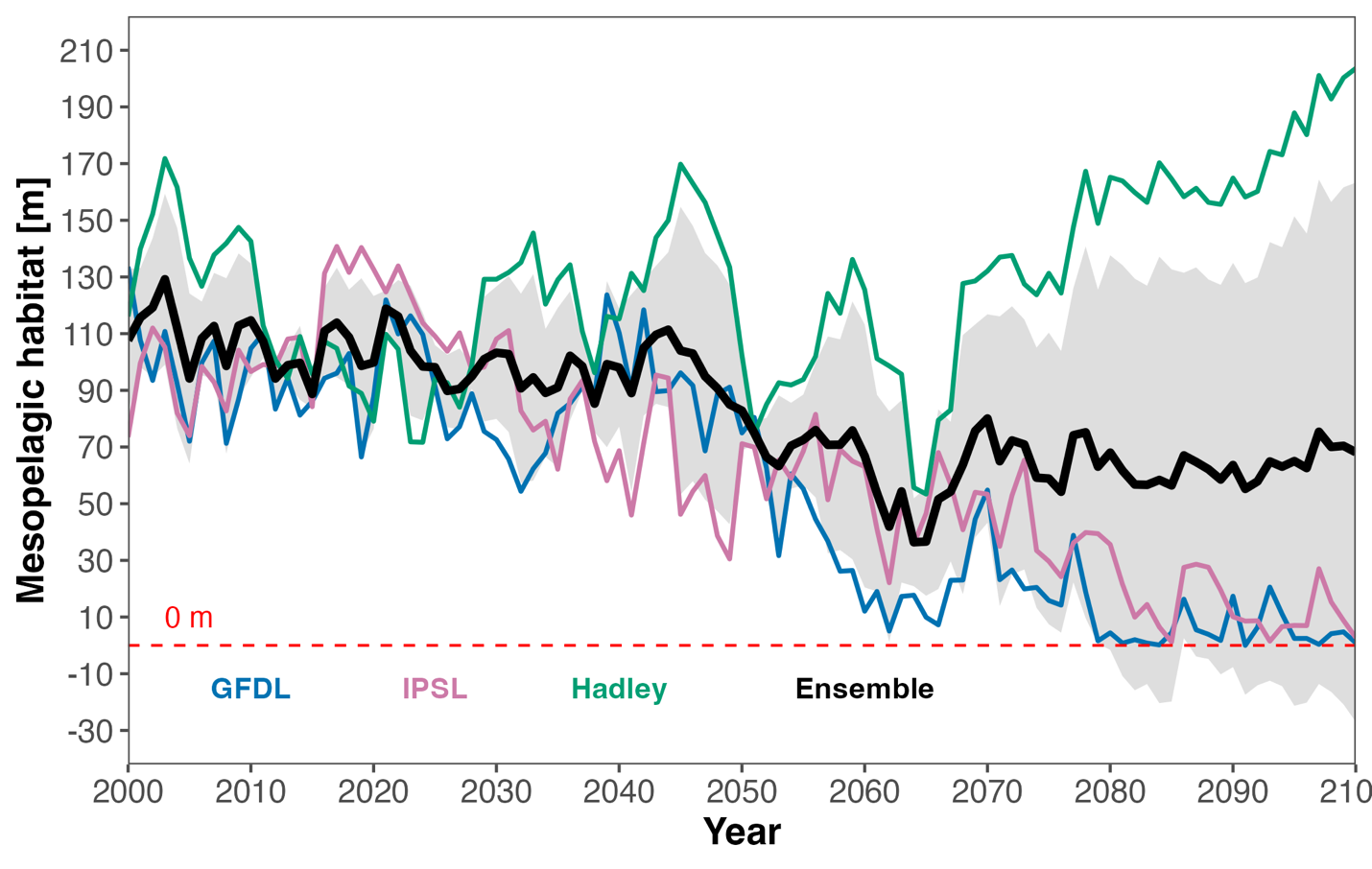


**c**

**Supplementary Figure 2** The ensemble mean (black), ensemble spread (grey, calculated as the standard deviation between the 3 ensemble members) and individual ensemble members ROMS-GFDL (blue), ROMS-IPSL (pink) and ROMS-HADL (green) for a. dissolved oxygen concentrations ($mmol/m^{3})$ at 300 m, b. Light intensity at a depth of 150 m (W/$m^{2}$) and c. vertical extent of mesopelagic habitat (m).


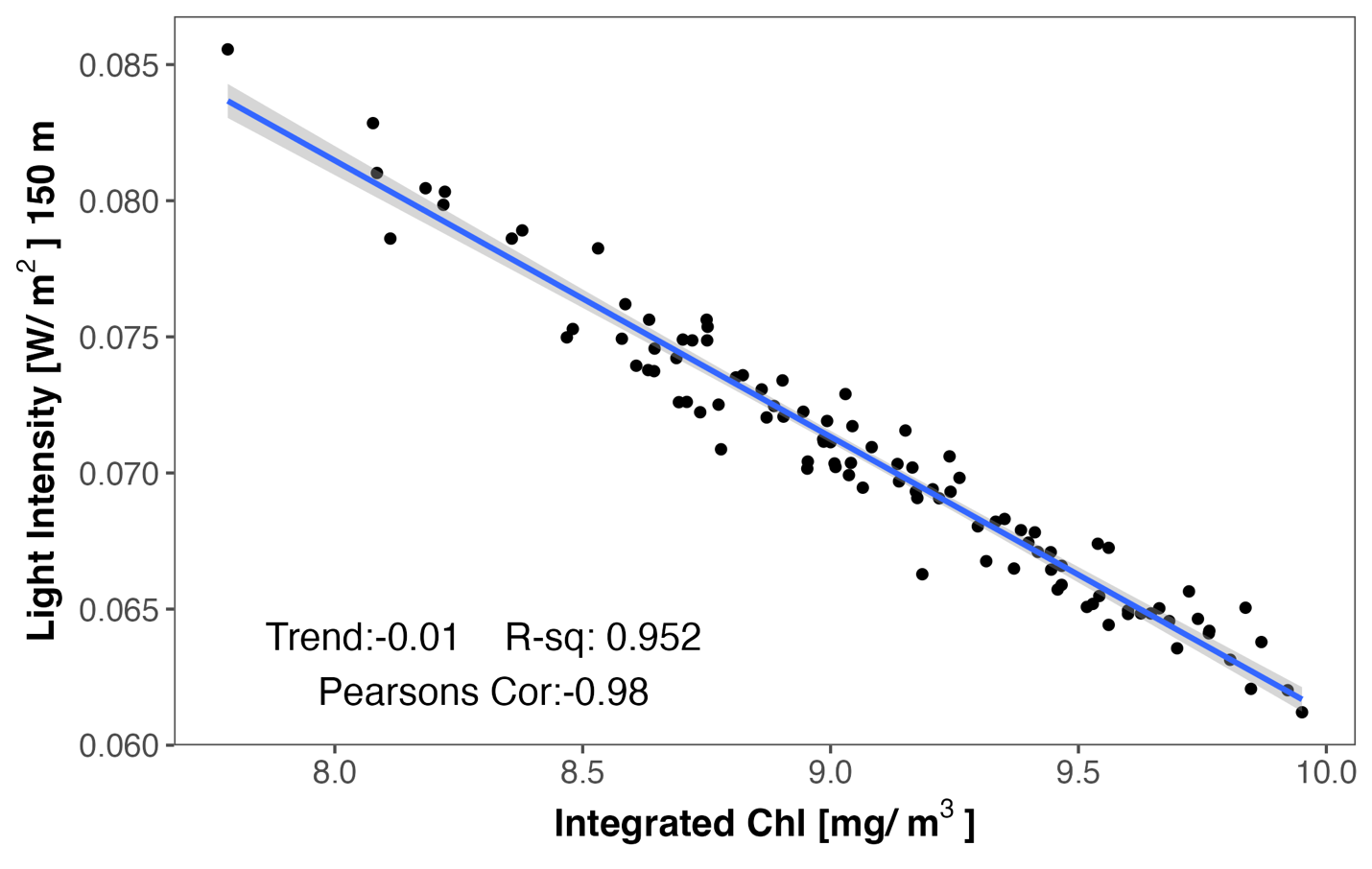


**Supplementary Figure 3** Correlation between spatially averaged ensemble means of depth integrated chlorophyll in the upper 100 m of the water column ($mmol/m^{3}$) and light at 150 m $(W/m^{2}$)
